# Genome-wide Profiling of Histone Lysine Butyrylation Reveals its Role in the Positive Regulation of Gene Transcription in Rice

**DOI:** 10.1186/s12284-019-0342-6

**Published:** 2019-11-27

**Authors:** Shuai Liu, Guanqing Liu, Peifeng Cheng, Chao Xue, Yong Zhou, Xu Chen, Lu Ye, Zhongying Qiao, Tao Zhang, Zhiyun Gong

**Affiliations:** 1 Jiangsu Key Laboratory of Crop Genetics and Physiology / Jiangsu Key Laboratory of Crop Genomics and Molecular Breeding/ Jiangsu Co-Innovation Center for Modern Production Technology of Grain Crops, Agricultural College of Yangzhou University, Yangzhou, 225009 China; 2 Joint International Research Laboratory of Agriculture and Agri-Product Safety / Key Laboratory of Plant Functional Genomics, the Ministry of Education of China, Yangzhou University, Yangzhou, 225009 China; 3 Suzhou Academy of Agricultural Sciences, North of Wangting Town, Suzhou, 215128 China

**Keywords:** *Oryza sativa*, Histone modification, Lysine butyrylation (Kbu), Transcriptional regulation, ChIP-seq

## Abstract

**Background:**

Histone modifications play important roles in growth and development of rice (*Oryza sativa* L.). Lysine butyrylation (Kbu) with a four-carbon chain is a newly-discovered histone acylation modification in rice.

**Main Body:**

In this study, we performed chromatin immunoprecipitation sequencing (ChIP-seq) analyses, the result showed that major enrichment of histone Kbu located in genebody regions of rice genome, especially in exons. The enrichment level of Kbu histone modification is positively correlated with gene expression. Furthermore, we compared Kbu with DNase-seq and other histone modifications in rice. We found that 60.06% Kub enriched region co-located with DHSs in intergenic regions. The similar profiles were detected among Kbu and several acetylation modifications such as H3K4ac, H3K9ac, and H3K23ac, indicating that Kbu modification is an active signal of transcription. Genes with both histone Kbu and one other acetylation also had significantly increased expression compared with genes with only one acetylation. Gene Ontology (GO) enrichment analysis revealed that these genes with histone Kbu can regulate multiple metabolic process in different rice varieties.

**Conclusion:**

Our study showed that the lysine butyrylation modificaiton may promote gene expression as histone acetylation and will provide resources for futher studies on histone Kbu and other epigenetic modifications in plants.

## Background

Epigenetic regulation of gene expression is an intricate process that does not involve a change in DNA sequence. Epigenetic marks, such as DNA methylation, histone modification and non-coding RNA have significant effects on regulating transcription. Among these, post-translational modifications (PTMs) of histones that may transform the chromatin state are essential for gene expression. With the development of new biotechnique and updates to PDBs (protein data banks), increasing numbers of histone PTMs have been identified; for example, lysine acylations modifications have various forms, such as acetylation (Tropberger et al. 2013), butyrylation (Zhang et al. 2009), propionylation (Zhao and Jensen 2009), crotonylation (Tan et al. 2011), methylation (Peach et al. 2012), malonylation (Xie et al. 2012), succinylation (Zhang et al. 2011), 2-hydroxyisobutyrylation (Dai et al. 2014), and β-hydroxybutyrylation (Liu et al. 2019). These acylation modifications always mark lysine with different hydrocarbon chain lengths and hydrophobicity or charge (Azevedo and Saiardi 2015).

Lysine acetylation, which is one of the most studied modifications, is essential for the control of gene expression. Previous research has indicated that there are complex interactions between lysine acetylation and transcription factors, such as enhancers, silencers, and promoters (Perillo et al. 2008; Bannister and Kouzarides 2011). The level of histone acetylation is maintained by histone acetyl-transferases (HAT) and histone-deacetylases (HDAC) (Zhao et al. 2014). Also, histone acylation homeostasis can be maintained by different histone complexes associated with these two enzymes, such as crotonylation, butyrylation, and propionylation (Ogryzko et al. 1996; Chen et al. 2007; Kaczmarska et al. 2017; Sabari et al. 2018). Human sirtuins, which have homology to the yeast sir2 histone deacetylase, have both deacetylase and deacylase activities (Ogryzko et al. 1996; Cheng et al. 2009; Sabari et al. 2017).

Lysine butyrylation (Kbu) is a novel PTM that is found widely in histone and non-histone proteins (Chen et al. 2007). Kbu has been identified in multiple cell types in animals, plants, and fungi, suggesting that Kbu is evolutionarily conserved (Goudarzi et al. 2016; Lu et al. 2018). Butyrylation is an acylation modification similar to crotonylation, with a four-carbon chain in the planar orientation (Flynn et al. 2015). Interestingly, the histone Kbu marks active gene TSSs and directly stimulates transcription (Goudarzi et al. 2016). In addition, H4K5bu can prevent binding of the bromodomain testis-specific gene (BRDT) (Goudarzi et al. 2016). Moreover, H4K5bu and H4K8bu are related to delayed histone removal in spermatogenic cells (Goudarzi et al. 2016). Recently, the double plant homeodomain finger (DPF) of the lysine acetyltransferase MORF (the monocytic leukemia zinc-finger protein-related factor) was shown to be a reader of global histone H3K14 acylation that can bind H3K14bu to form a recruitment and stabilization MORF-DPF-H3K14bu complex at promoters of target genes (Klein et al. 2017). Because they are photoautotrophic organisms, plants are significantly different from mammals with respect to their primary metabolic processes. The relationships between butyrylation and gene expression in the interactions with primary metabolism in plants are less known at present.

Rice (*Oryza sativa* L.) is a model monocot species that plays a fundamental role in plant genome research (Shi et al. 2015). Several protein modifications have been identified in rice, such as methylation (Cheng et al. 2018), acetylation (Xue et al. 2018), and crotonylation (Liu et al. 2018). Recently, butyrylation, was identified by Lu et al. (2018) as an active modification mark that regulates gene expression in the rice cultivar ‘DongJin’ (DJ) (Lu et al. 2018). Therefore, we performed additional experiments and a combined public data analysis to identify histone Kbu in the *japonica* rice cultivar ‘Nipponbare’.

We confirmed that Kbu is present in histones and non-histone proteins in rice using biological experiments. We also profiled the genome-wide distribution of the Kbu modification by ChIP-seq analysis with a pan anti-Kbu antibody. In addition, we compared Kbu with 12 other histone modifications and DHS in rice. In brief, our research will enlarge the discovery of the biological functions of histone lysine butyrylation in rice.

## Results

### Genome-wide Profiling of Histone Kbu in Rice

**Fig. 1:**
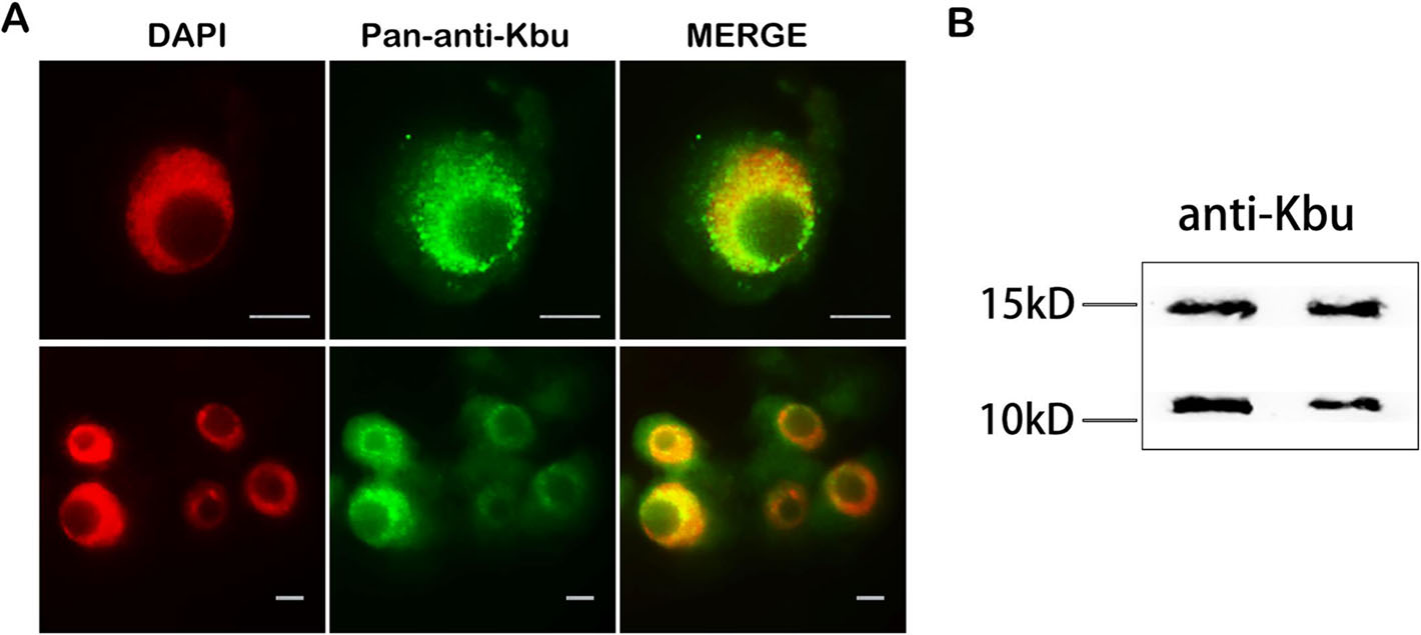
An overview of Kbu modifications in rice. **a** lysine butyrylation was detected in the nucleus and cytoplasm of two-week-old rice root cells by immunofluorescence using an anti-Kbu antibody (green), and the nuclei were stained with DAPI (red). Scale bars: 5 μm. **b** Western blot analysis of histones in 14-day-old rice seedling leaves with anti-Kbu antibody

Histone Kbu has been identified previously in rice variety Dongjin by mass spectrometry (Lu et al. 2018). To further confirm the existence and distribution of Kbu, we performed Western blotting (WB) and immunofluorescence (IF) analysis using a pan anti-Kbu antibody in rice variety Nipponbare. We observed that butyrylated proteins were clearly distributed in the nuclei and cytoplasm by IF (Fig. 1a). In addition, WB analysis of the core histones revealed that the Kbu signals co-migrated with bands of approximately 15 kD and 10 kD, respectively, which correspond to the sizes of histones H3 and H4 (Fig. 1b). From these analyses, we tentatively conclude that Kbu is present in rice histones.

We next investigated the biological function of histone Kbu in rice. ChIP-seq analysis was performed using the pan anti-Kbu antibody in seedlings. To obtain the genomic distribution of Kbu in rice, we constructed the ChIP-seq libraries on the Illumina HiSeq 2500 instrument with two biological replicates. A total of 25.7 million paired-end reads were obtained (Table 1), most of which (> 88%) mapped to the rice reference genome. We found that 81.99% of the peak reads were shared between the two libraries, indicating that ChIP-seq data is reliable and reproducible. The common peaks (21,202) were then further analyzed as histone Kbu-enriched reads in rice seedlings (Table 1).

**Table 1:** Summary of ChIP-seq data

Libraries	Reads numbers	Mappable reads	Peaks	Common peaks
Kbu(pan-antibody) replicate 1	97,003,162	86,035,363(88.69%)	30,395	21,202
Kbu(pan-antibody) replicate 2	101,530,760	91,283,544(89.91%)	31,764	

To examine the reliability of the analysis results by ChIP-seq, one peak site and one non-peak site were randomly chosen from loci on each of the 12 chromosomes for ChIP-qPCR validation. Peak with qPCR2 values > 1 represents Kbu-enrichment. Nine out of 12 peaks showed enrichment of Kbu (Table 2). On the contrary, only two of the non-peaks showed enrichment of Kbu. Thus, the ChIP-qPCR results were generally consistent with analysis of the ChIP-seq libraries.

**Table 2:** Confirmation of Kbu peak sites and non-peak sites by quantitative PCR

	Genomic region	Forward primer	Reverse primer	qPCR1	qPCR2
Peak sites	LOC_Os01g02960	GACATGGTCACTGTCCCCAG	GATGCCATCTTCGTTGACGC	0	1.23249
	LOC_Os02g10180	AATTACTTGCCACCGCCAGA	ATAGTCACCCTCCGCTTCCT	0	1.475
	LOC_Os03g06210	CGCGTGTACCGACGAGAAAA	TGTTGCCTACGTTCTCCACC	0	1.807216
	LOC_Os04g37580	TGCGTTGGGAATCAAACCCT	TCATCGTGGCTGGCTTATGG	0	1.052905
	LOC_Os05g04950	GGGGACATGTTGAGTGAGGG	CAACAACTGGCTGGGCAATC	0	1.030004
	LOC_Os06g06410	GAGCAAGGGCCCTAAGTTCG	TAGGCACTCACACATTCCGC	0	0.794
	LOC_Os07g17220	CAAAATTGCGAAGAACTGCCG	CCAGGCTCCCATATCCCTGAA	0	0.568885
	LOC_Os08g08205	GCCAGGTGAGATTAGGCCAG	TTCCTGACAAATGCCTGCCA	0	1.656729
	LOC_Os09g28310	TGTCCCACCCTAGAGACCAG	AGCTAGTCATCAGGCAGGTTG	0	1.656729
	LOC_Os10g28254	TCCGATTAGGTTGGCTATATTCAT	TGAAGCACTTCCACACAAGT	0	0.382959
	LOC_Os11g26130	GCCACTGTGTGAACCGACTA	AGGGTTGCCCTTGCGAATTA	0	0.401704
	LOC_Os12g43750	TTCCAAACCAACCAACTCCCT	TCCCACGAGAACATCACGGT	0	1.184018
Non-peak sites	LOC_Os01g01320	TGGTGCACAATGCTGAGACT	CCAGTTTCAGAGTAGTTGATGGC	0	−1.34071
	LOC_Os02g38870	AGGAGGAAGAGGGGCCTAAG	TCGTGTCCATCTCCTCGTCT	0	−0.1424
	LOC_Os03g02650	ATGGGCTTACGGGTGCATAG	CCTATCTCGCATACGTGCCG	0	0.419453
	LOC_Os04g02030	GAGCAAGGTCCTGGTCACAA	GGCCATCAAGACTCACAGCA	0	−0.32465
	LOC_Os05g25510	TCTCAGTGGTGGGGAAGGAT	ACCATTGCTCACCTCAAGCA	0	−0.57654
	LOC_Os06g02930	GGTCATGAAGGTCATCCACGG	CCGTGATGTTGGGCAAGTAGA	0	1.961737
	LOC_Os07g37370	GTGAGGGTGAGAGGGGAAAG	AAATTAGCTCCCGGACTGGC	0	0.739465
	LOC_Os08g23640	CAACGACATCGTGCTCGC	GCGACGCCGTACCTGAAG	0	−0.09366
	LOC_Os09g39170	AACCCATCATCACGGTGGAC	AGAGATGGGCTGCTGGTAGA	0	1.608844
	LOC_Os10g01590	ATGACCACAAAACGGTTCGG	GATTGACTCGCGCTATGCAG	0	−0.97679
	LOC_Os11g02810	GGATTTCGCGATGGGGATTC	AAGTGGTTCGCAACGCAATC	0	0.866769
	LOC_Os12g43220	GGTGCTAGGAATCGACCCAA	CTGCCATCACCAAGGGGAAT	0	−0.68791

1Normalized C(t) of input DNA

2Normalized ⊿C(t) of ChIP DNA

### Histone Kbu is Related to Gene Expression in Rice

**Fig. 2:**
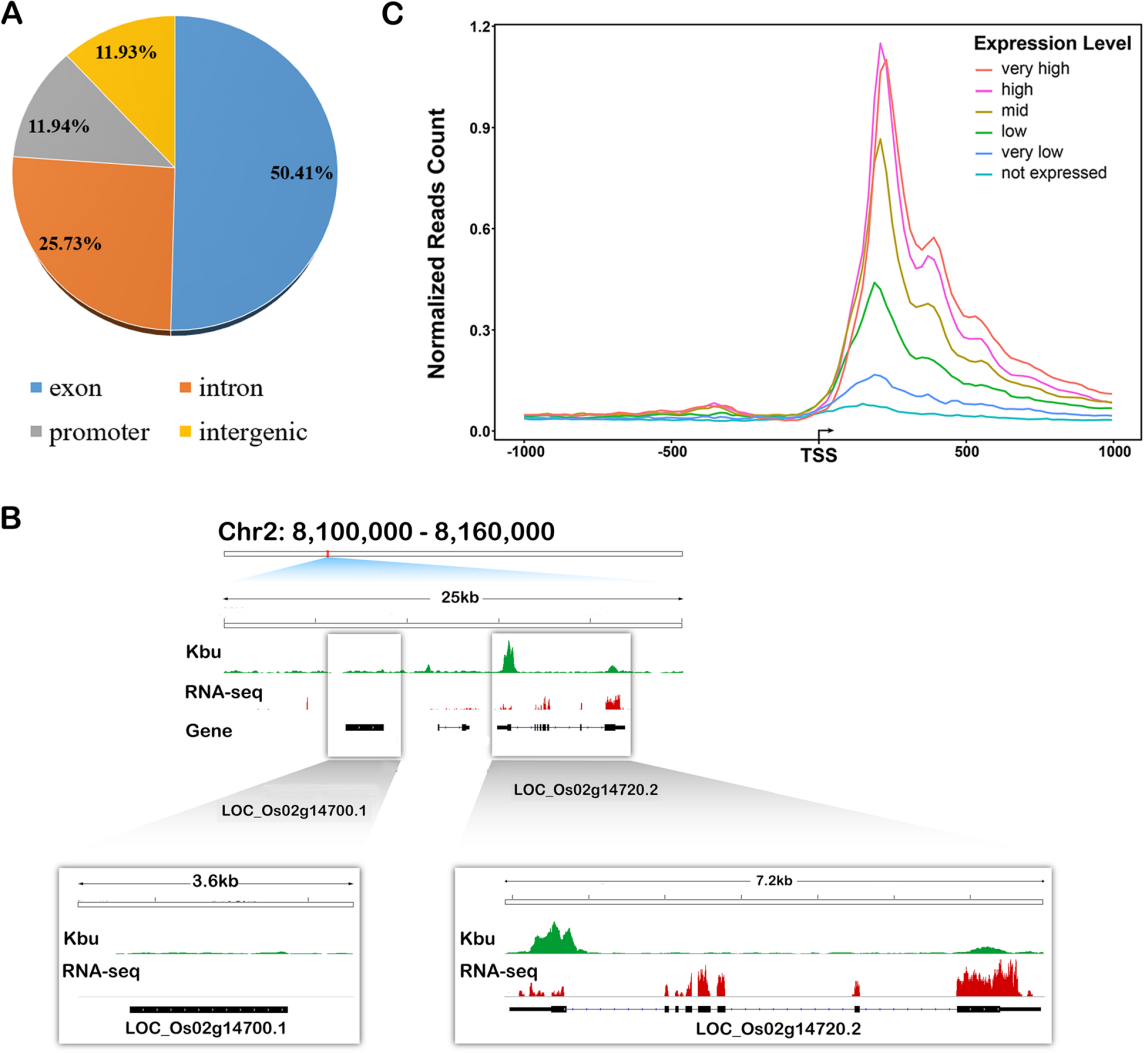
The genomic distribution of histone Kbu-enriched regions in rice. **a** Genome-wide distribution of histone Kbu in the rice genome. The promoter regions are defined as the 1 kb of DNA sequence directly upstream of the gene transcription start site (TSS). **b** Mapping of Kbu sites, RNA-seq reads and gene loci in a 60,000 base pair region on Chr2:8,100,000 – 8,160,000. **c** Distribution of Kbu density around differentially expressed genes. The Kbu modification level was calculated by the number of reads per kilobase in the mapped genomic region. The arrow indicates the direction of transcription from the transcription start site (TSS). The rice genes were divided into six categories based the expression level from the top 20% to the bottom 0% based on published RNA-seq data from seedlings of the same rice cultivar at the same stage of development (Zhang et al. 2012)

According the ChIP-seq analysis, the genomic distribution of histone Kbu-enriched regions was determined, which can be divided into four categories. The peaks covered a large proportion of the genebody regions (76.14%), especially in exons (50.41%), while only 25.73% were found in the intron regions (Fig. 2a). In addition, 11.93% and 11.94% of the peaks in the Kbu regions were located in the promoter (1 kb upstream of TSS) and intergenic regions, respectively (Fig. 2a). Most of the Kbu peaks in the exons were mapped to the coding sequences (38.77% of 21,202 Kbu regions) and the 5′-UTR (6.8% of 21,202 Kbu regions), while only 2.54% were located in the 3′-UTR. In Dongjin (Lu et al. 2018), about 27,665 peaks were identifed with MACS2. Campared to similar analysis in Dongjin, 84.70% peaks (17,958 out of 21,202) in Nipponbare are shared with Dongjin (Table 3).

**Table 3:** Summary of Kbu-related genes and peaks of two rice varieties (Nipponbare and DongJin)

	Nipponbare	DongJin
Number of peaks	21,202	27,665
Common peaks	17,958	17,958
Kbu-related genes	14,808	19,355
Common Kbu-related genes	12,559	12,559
Specific Kbu-related genes	2249	6796
Kbu-related expressed gene	11,984	14,151
Common expressed gene	9961	9961

We next investigated the relationship between gene expression and histone Kbu. Among total 39,045 non-TE genes in rice, 14,808 genes (37.93%) are marked by histone Kbu. In these genes, 80.93% genes (11,984) are expressed, with FPKM> = 1 (Wu et al. 2011). For example, the peaks of Kbu ChIP-seq located at expressed gene LOC_Os02g14720, rather than non-expression gene LOC_Os02g14700 (Fig. 2b). The results suggested that the sites of histone Kbu were enriched principally in expressed genes. In addition, we found genes with generally higher expression levels associated with higher histone Kbu density (Fig. 2c) In Dongjin (Lu et al. 2018), the peaks (27,665) were identifed in 19,355 genes, in which 14,151 genes (73.11%) are expressed in Dongjin, with FPKM> = 1, while 11,984 genes (80.93%) are expressed in Nipponbare. Nine thousand nine hundred sixty-one genes are shared between two cultivars (Table 3). These results show that histone Kbu has a high correlation with gene expression in different cultivars of rice.

### Concurrence among Histone Kbu, other Histone Modifications, and DNase-hypersensitive (DH) Sites

**Fig. 3:**
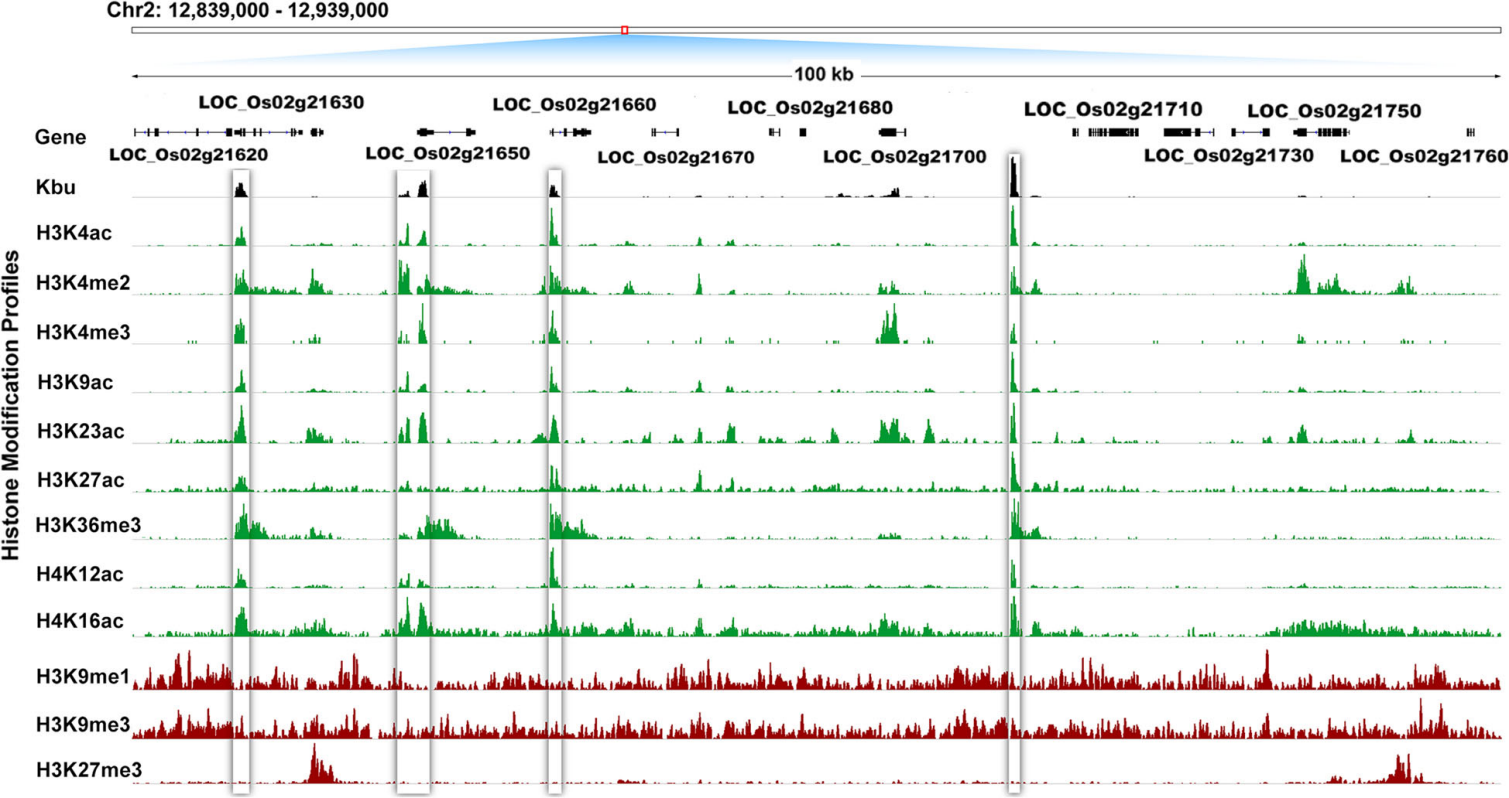
Gene transcription in the rice genome and the genomic distribution of histone Kbu and other modifications. Visualization of the Kbu sites and 12 other histone modifications against 10 gene loci in a 100,000 base pair region on Chr2:12,839,000 – 12,939,000

**Fig. 4:**
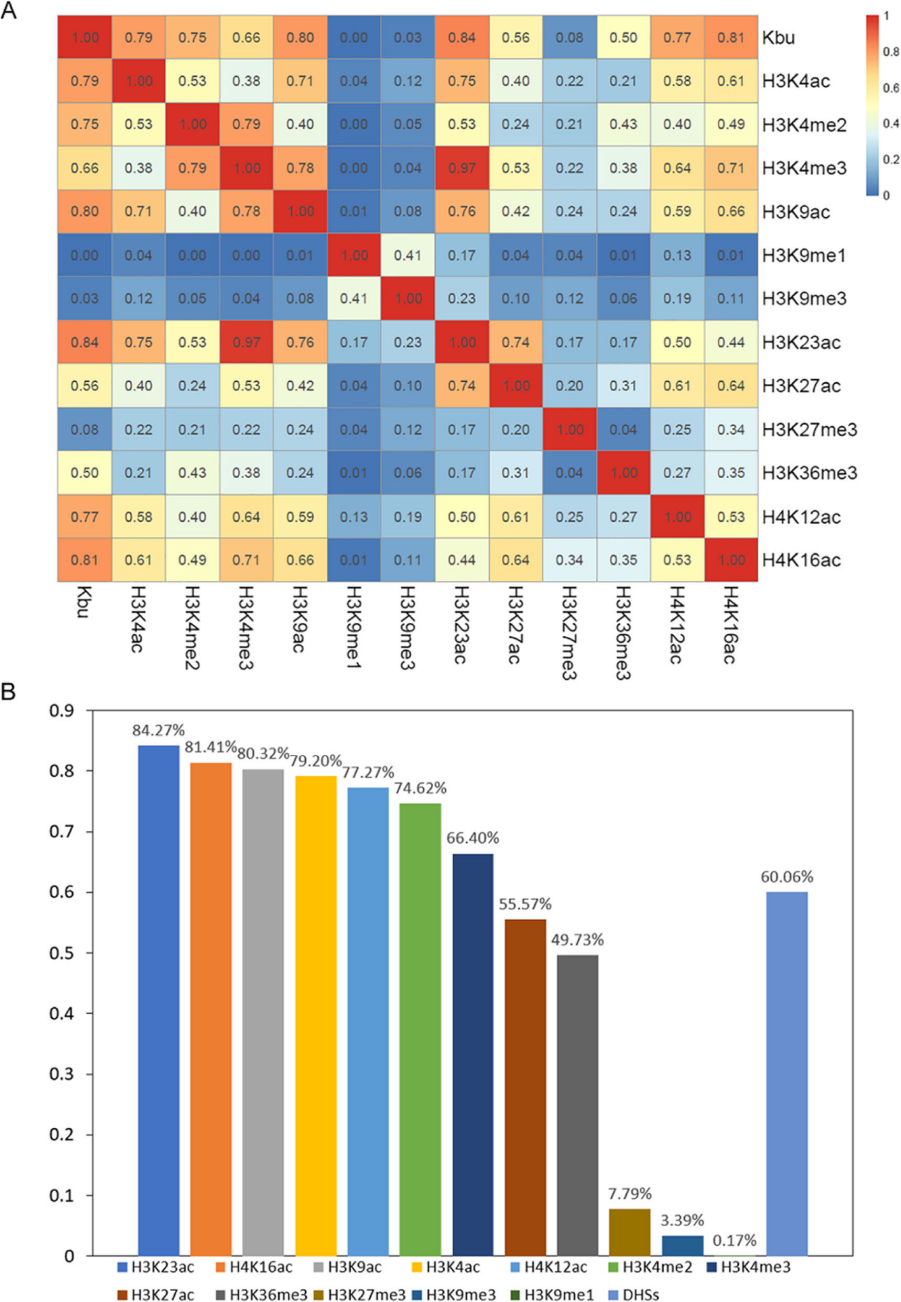
The percentage of histone Kbu-enriched regions that overlap with regions enriched with 12 other histone modifications. **a** Heatmap **b** Histogram

**Fig. 5:**
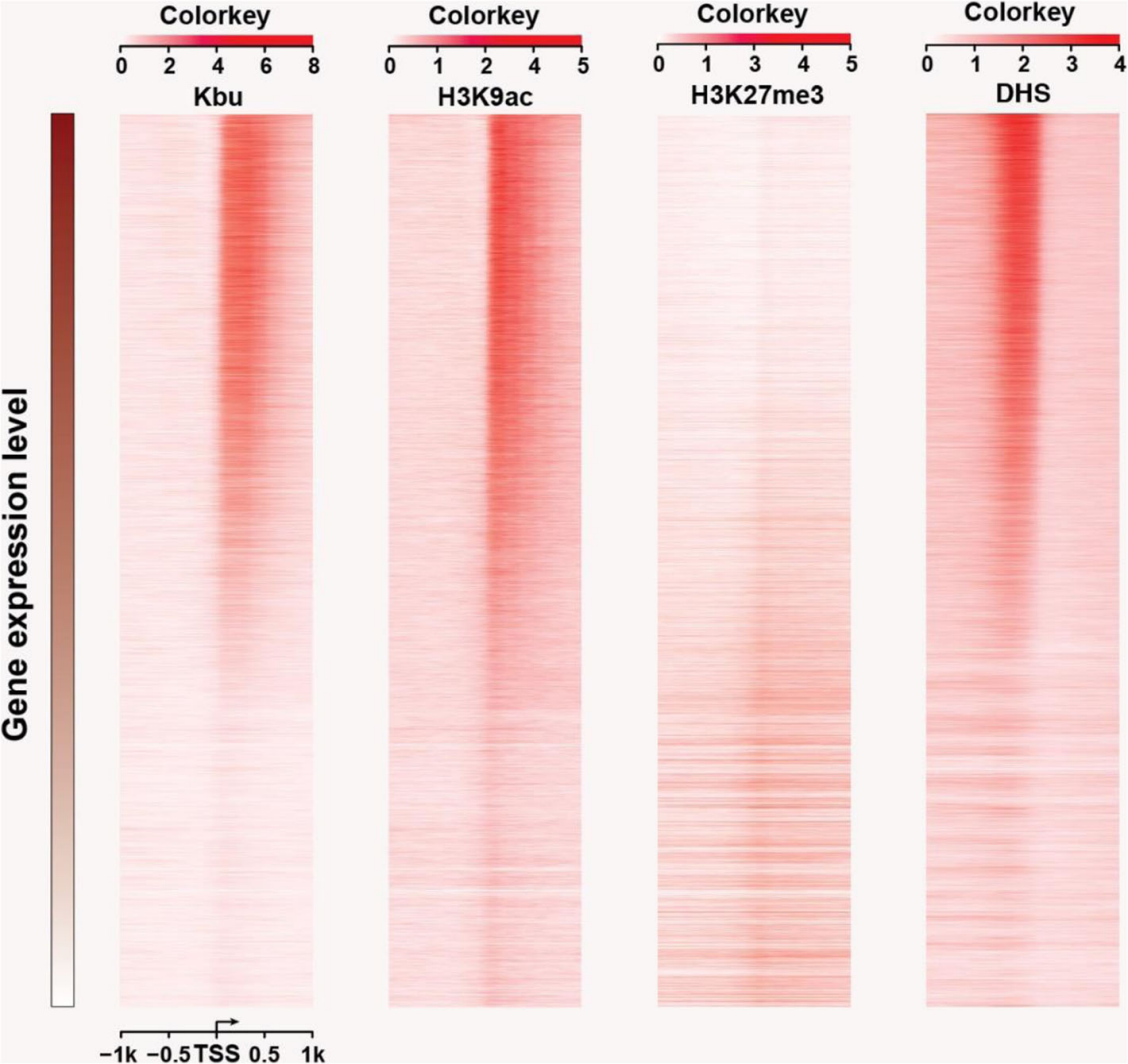
Heatmaps of histone Kbu, H3K9ac, H3K27me3, and DHS in gene regions and intergenic regions. The DNA regions 1 kb upstream and 1 kb downstream of the transcription start sites were analyzed. Density was calculated by the number of reads per kilobase region per million mapped reads

Interestingly, our results showed that histone butyrylation can facilitate gene expression in rice, similar to some canonical histone modifications such as H3K4ac, H3K9ac, and H3K36me3 (Fang et al. 2016a). Therefore, we mapped public data for 12 histone modifications (H3K4ac, H3K9ac, H3K23ac, H3K27ac, H4K12ac, H4K16ac, H3K4me2/3, H3K36me3, H3K9me1/3, and H3K27me3) in rice (He et al. 2010; Zhang et al. 2012; Lu et al. 2015; Fang et al. 2016b), and analyzed the concurrence between histone Kbu and these histone modifications (Fig. 3). The results showed that histone Kbu is usually enriched in regions shared with all other active lysine marks, including H3K4ac, H3K9ac, H3K23ac, H3K27ac, H4K12ac, H4K16ac, H3K4me2/3, and H3K36me3. Statistical analysis of the concurrences revealed large proportions of overlap between histone Kbu and H3K23ac (84.27%), H3K4ac (81.41%), H3K9ac (80.32%), and H3K4ac (79.20%) (Figs. 4 and 5). We also found that histone Kbu was not located in regions associated with repressed marks like H3K9me1 (0.17%), H3K9me3 (3.39%), and H3K27me3 (7.79%). This suggests that histone Kbu is a transcription-activating modification.

In the mouse, 35% H3K14bu peaks cover the promoter-TSS regions (Kebede et al. 2017). Here, we found 23.87% histone Kbu modifications were enriched in promoter and intergenic regions in rice. We next investigated the relationship between histone Kbu and DNase I hypersensitive sites (DHSs), because these sites harbor cis-regulatory elements in open chromatin. More than half of the histone Kbu peaks overlapped with DHSs in the rice genome (Zhang et al. 2012), and 60.06% of the peaks in intergenic regions co-located with DHSs (Figs. 4b and 5). These results indicate that histone Kbu can be an active mark and may recruit transcriptional regulators to facilitate gene transcription.

### Histone Kbu Combined with Histone Acetylation Facilitates Transcription

**Fig. 6:**
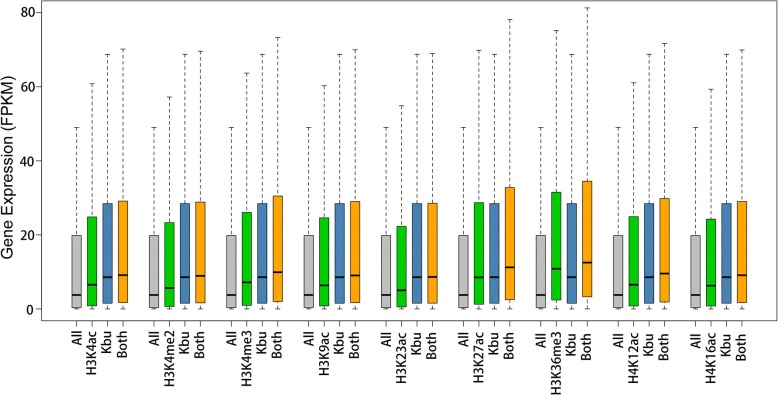
Comparisons of the expression of genes associated with different combinations of histone modifications. The non-TE gene expression values (FPKM: Fragments per Kilobase Million) of each combination are indicated by box plots. All: all rice genes. Kbu: all genes with Kbu modifications. Both: genes with both Kbu and one of the other nine modifications as shown. The asterisks (*) indicate a significant difference between the pairwise combinations (*p* < 2.2e-16, Kolmogorov-Smirnov test)

Our previous results showed that histone Kbu can promote gene expression, and share similar locations with other histone acetylation modifications. We therefore wondered whether genes with both histone Kbu and acetylation modifications show higher levels of gene expression. We classified genes into three categories: genes with Kbu or other modifications only, and genes with both Kbu and any of another nine histone modifications (Fig. 6). The results of this analysis showed that genes with histone Kbu had significantly higher expression compared to genes without histone Kbu. Genes with both histone Kbu and one other acetylation also had significantly increased expression, suggesting that histone Kbu participates in transcriptional regulation, and also collaborates with other histone acetylation to facilitate gene expression in rice.

### Putative Functions of Genes Associated with Histone Kbu

**Fig. 7:**
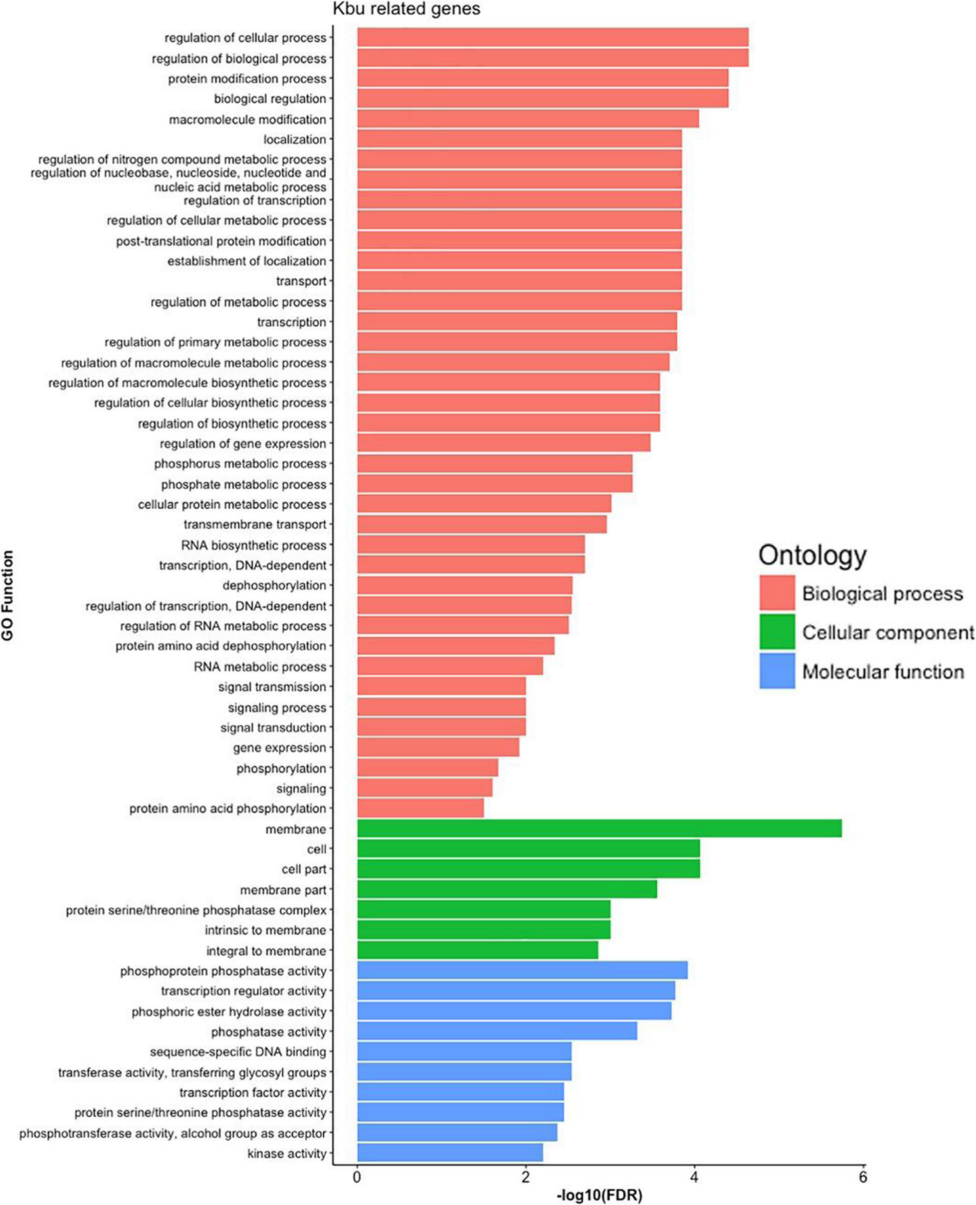
Functional annotation of the lysine butyrylome. Representative GO annotations of histone Kbu sites for genes in the three primary GO categories “biological process”, “molecular function”, and “cellular component”. Gene ontology (GO) analysis was performed on 1480 genes that showed significant histone Kbu enrichment out of 14,808 Kbu-related genes in this study

To gain an intial understanding of the putative functions of genes associated with histone Kbu, we identified 1480 genes that showed significant histone Kbu enrichment out of 14,808 Kbu-related genes and performed GO analysis on them. GO enrichment analysis showed that most of these genes participate in many important biological processes, such as regulation of cellular and biological processes, protein modification transport, transcription, signal transduction, and gene expression, etc. Ths single GO term containing the most genes is “membrane” in the major GO category “Cellular Component” (Fig. 7). Furthermore, these genes take part in many molecular functions related to phosphorylation. All of these results indicate that histone Kbu may participate in epigenetic regulation involving phosphorylation.

Compared to the Kbu-related genes in DongJin (Lu et al. 2018), although there are highly similarly between Nipponbare and DongJin, there are still many different genes. We further conduct the GO analysis on 2249 and 6796 specific Kbu-related genes in Nipponbare and Dongjin, respectively (Table 3). Genes with histone Kbu in Nipponbare are enriched in translation, transport and localization process, while in Dongjin, gene with such histone modification participates in transcription, binding process and stress-response. Nevertheless, both in Nipponbare and Dongjin, these genes can regulate multiple metabolic processes. Hence Kbu is important for growth and development of rice.

## Discussion

Recently, Lu et al. (2018) identified four rice histone lysine butyrylation sites (H3K14bu, H4K12bu, H2BK42bu, and H2BK134bu) using LS-MS/MS in the rice cultivarDongJin. Their results showed that Kbu is enriched in the 5′ regions of expressed genes, and 26,769 Kbu-marked genes were identified. In this study, 21,202 histone Kbu-marked peaks also appeared to be mainly in the 5′ regions, TSS regions, and exons. The peaks covered a large proportion of the genebody regions, especially in exons. Most of the Kbu peaks in the exons were mapped to the coding sequences and the 5′-UTR. This distribution is similar to that of histone Kbu in Dongjin (Lu et al. 2018). Meanwhile, it is similar to that of histone H3K4me2/3 in rice (Du et al. 2013). However, in mouse, H3K14bu is mostly enriched in introns and promoter-TSS regions, and more than two-thirds of the peaks covered these regions (Kebede et al. 2017). These results suggest that histone Kbu may regulate gene expression via different mechanisms in plants and mammals. In addition, we found genes with generally higher expression levels associated with higher histone Kbu density, as was found for H3K14bu in mouse and Kbu in other rice variety (Kebede et al. 2017; Lu et al. 2018). These results show that histone Kbu has a high correlation with gene expression in eukaryote.

Lu et al. (2018) showed that Kbu is an active mark, but data for only six histone modifications was used to compare with Kbu. In addition, histone Kbu seems to contribute to the H3K9ac-marked active chromatin state and to balance genes under stress. In this study, we also found that the enrichment level of Kbu is proportional to gene expression. Moreover, we integrated our data with 12 public histone modification data, including H3K4ac, H3K9ac, H3K23ac, H3K27ac, H4K12ac, H4K16ac, H3K4me2/3, H3K36me3, H3K9me1/3, and H3K27me3 (He et al. 2010; Zhang et al. 2012; Lu et al. 2015; Fang et al. 2016b) and also verified the large proportion of Kbu sites overlapping with H3K9ac. These results abundantly showed that histone lysine butyrylation is consistent with active histone modifications such as H3K4ac, H3K9ac, H3K23ac, H3K27ac, H4K12ac, H4K16ac, H3K4me2/3, and H3K36me3. Interestingly, H3K36me3 is similar only to Kbu and H3K4me3, which suggests that H3K36me3 may have similar functions to Kbu and H3K4me3. In addition, 60% of the sites overlap with DHSs, suggesting that Kbu is related to cis-regulatory DNA elements in rice. Our analysis will enlarge a general understanding of epigenetic regulation of transcription via histone Kbu modification, and will enable investigation into the crosstalk between different histone modifications in plants.

It is important to identify and characterize histone modification enzyme systems in order to understand how histone modification is regulated. Some histone acylation is known to be associated with histone acetyltransferase and histone deacetyltransferase (Ogryzko et al. 1996; Chen et al. 2007; Kaczmarska et al. 2017; Sabari et al. 2018). Lu et al. (2018) confirmed that OsSRT2 possesses decrotonylase activity, but not debutyrylase activity. However, the p300 protein, which is an important histone acetyltransferase, also catalyzes histone butyrylation in human (Chen et al. 2007). In our study, we found that histone Kbu tends to co-localize with multiple active histone modifications, especially Kac. In a previous study, three p300 homologous genes were identified in a phylogenetic analysis in rice (Liu et al. 2018). However, it is presently unknown whether p300 catalyzes histone Kbu and, if so, how acetylation and butyrylation is regulated in rice. Additionally, the role of histone Kbu in the regulation of histone structure and function in rice requires further investigation.

## Materials and Methods

### Materials

Rice (*Oryza sativa*) cultivar ‘Nipponbare’ plants were germinated and grown in water without hormones under a 12 h/12 h photoperiod at 28 °C day/25 °C night with 70% humidity. Leaves and stem tissues of 14-day-old rice seedlings were used for histone protein extraction and ChIP-DNA isolation.

### Histone Protein Extraction

Histone proteins were extracted based on a previous method (Liu et al. 2018). Leaves and stem tissues of 14-day-old rice seedling were first ground to a powder in a mortar in the presence of liquid nitrogen. The powdered tissue was then mixed with extraction buffer and centrifuged. The pellet was mixed with nuclei lysis buffer for 30 min on ice, centrifuged again, and the supernatant was removed. The pellet was resuspended in 0.2 M HCl and incubated on ice for 1 h. The proteins were precipitated by addition of 100% TCA, recovered by centrifugation and washed with cold acetone. The sediment was redissolved in Protein Lysis Buffer by sonication and stored at − 80 °C.

### Western Blotting and Immunofluorescence Analyses

Western blotting was performed as previously described (Liu et al. 2018). Immunofluorescence analysis was performed using the method described by Gong et al. (Gong et al. 2009). The rice histone proteins were separated electophoretically in denaturing gels by SDS-PAGE (5%/12%). The antibodies used in this study were rabbit pan anti-Kbu antibody (1:5000; PTM BioLabs, HangZhou China, PTM-301) and rabbit anti-H3 antibody (1:10,000; PTM BioLabs, HangZhou China, PTM-1001); the goat secondary anti-rabbit antibody is conjugated with Alexa 488 (Invitrogen, A11008). Chromosomes were counterstained with DAPI dye (Vector Laboratories, H-1200).

### ChIP, ChIP-seq, and qPCR

ChIP experiments were performed using a pan anti-Kbu antibody (PTM BioLabs, PTM-301) following a published protocol (Nagaki et al. 2003). Chromatin fragments were obtained by incubation overnight with MNase and protein A-coated beads (GE17–1279-01; Sigma Aldrich). The ChIP-DNA fragments were used for library construction with the Illumina protocol and were then sequenced on the Illumina HiSeq 2500 instrument. ChIP-qPCR was performed using SYBR qPCR Master Mix (Vazyme, Q311–02/03) according to the procedure described by Mukhopadhyay et al. (Mukhopadhyay et al. 2008). Input-DNA was set as the control and the following thermocycling conditions were used: initial denaturation at 95 °C for 600 s, three-step amplification comprising 35 cycles of 94 °C for 15 s to 60 °C for 15 s to 72 °C for 30s. qPCR2 values were acquired by subtracting ChIP-DNA C(t) from Input-DNA C(t), while the threshold cycles of Input-DNA (qPCR1) were set to 0. Input DNAs were used for normalization in ChIP-qPCR. The primers used in ChIP-qPCR are given in Table 2.

### ChIP-seq Data Analysis

Raw data were cleaned by cutadapt v2.1 with illumina TruSeq adapter. Bowtie2 v2.3.5 was used for mapping clean data to rice reference genome Tigr 7 (Langmead and Salzberg 2012; Kawahara et al. 2013). Only unique mapped reads without mismatch were retain for further analysis. Aligned bam files were converted to bigwig format using in-house script and visualized with IGV v2.4.5. MACS2 v2.1.2 was used to call peak with parameter ‘callpeak -g 3.8e8 --broad’ (Zhang et al. 2008). BEDTools was then used to merge replicates and identify shared peaks among different histone modification (Quinlan and Hall 2010). Genes contain Kbu peaks were regarded as Kbu related genes.

Based on expression level, all genes were divided into 6 groups. 1 kb upstream and downstream of each gene TSS regions were split into 20 bp bins to plot histone Kbu profile. Percentage of shared peaks of histone modifications was drawn by R package pheatmap. Heatmaps of all histone modifications were plotted using ngsplot v2.6.3 (Shen et al. 2014). All data processing and analysis were performed by python or R.

### Gene Ontology (GO) Analysis

One thousand nine hundred thirty-six peaks identified by macs2 with fold enrichment greater than 10 and -log10 (qvalue) greater than 100 were regarded as high confident peaks. We selected 1480 high-confidence histone Kbu-associated genes for gene ontology analysis using the agriGO v2 database (http://systemsbiology.cau.edu.cn/agriGOv2/) (Tian et al. 2017). The significance of a particular GO assignment was calculated using the Fisher test and corrected by FDR with a 0.05 significance level.

## Data Availability

All data have been uploaded to the National Coalition Building Institute Gene Expression Omnibus with accession number SRR8731856, SRR8735310. BioProject: PRJNA527145. Public histone modification data used in the article are as follows: H3K4ac, H3K9ac, H3K9me1, H3K9me3, H3K27ac and H3K27me3 were downloaded from GEO databases GSE79033 (Fang et al. 2016b). H3K4me2, H3K36me3 and H4K12ac were downloaded from GSE26733 (Zhang et al. 2012). H3K4me3 was downloaded from GSE19602 (He et al. 2010). H3K23ac and H4K16ac were downloaded from GSE69426 (Lu et al. 2015). Rice RNA-seq data were downloaded from GSE33265 (Wu et al. 2011).

## Abbreviations

BRDT: Bromodomain testis-specific gene
ChIP-seq: Chromatin immunoprecipitation sequencing
DHSs: DNase I hypersensitive sites
DJ: DongJin
DPF: Double plant homeodomain finger
GO: Gene Ontology
HAT: Histone acetyl-transferases
HDAC: Histone-deacetylases
IF: Immunofluorescence
Kbu: Lysine butyrylation
MOR: Monocytic leukemia zinc-finger protein-related factor
PTMs: Post-translational modifications
WB: Western blotting
